# Rachel Perkins

**DOI:** 10.1192/bjb.2022.41

**Published:** 2022-10

**Authors:** Abdi Sanati

**Figure:**
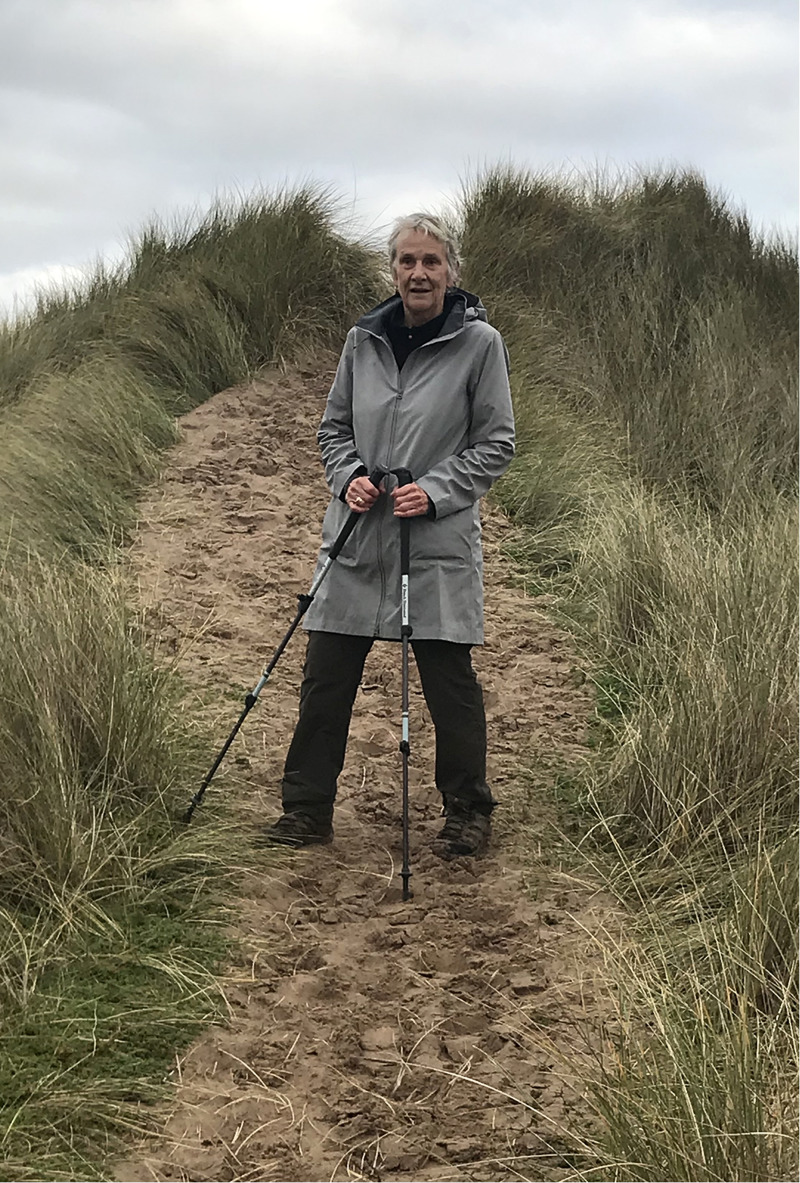

With a background in clinical psychology, Rachel Perkins worked in NHS mental health services for over 30 years, including 7 years as a Clinical Director and 5 years as Director of Quality Assurance and User Experience at South West London and St George's Mental Health NHS Trust. She now holds many roles: senior consultant with ImROC (Implementing Recovery through Organisational Change), which involves a range of consultancy, training and service development initiatives with statutory and voluntary organisations that wish to develop more recovery-focused services; non-executive director of Health and Employment Partnerships; non-executive director of Recovery Focus and Chair of its Working Together Committee; member of IPS Grow's Expert Forum; and Chair of IPS London Network.

She lives and works with a long-term mental health condition and in 2010 was voted Mind Champion of the Year and awarded an OBE for services to mental health. From 2011 to 2013 she was Chair of Equality 2025, the UK cross-government strategic advisory group on disability issues, and until recently chaired the Equality and Human Rights Commission (EHRC) Disability Advisory Committee and co-edited the journal *Mental Health and Social Inclusion*. She has written and spoken widely about recovery and social inclusion for people with mental health conditions, set up the first UK Recovery College and has pioneered the UK development of programmes to help people with mental health difficulties to access employment based on the individual placement and support (IPS) approach, including one designed to increase employment opportunities within mental health services for people who have themselves experienced mental health problems. In 2009 she was commissioned by the Secretary of State for Work and Pensions to lead an independent review into how government might better support people with mental health problems to gain work and prosper in employment.^1^

**Good afternoon Dr Perkins. You have been involved in the recovery model for about 20 years. What do you think of its state now, in 2022?**

I'm not sure I would use the term recovery model to start off with. For me recovery is about the challenge facing anyone with mental health issues, or indeed anyone who has experienced something that's knocked the bottom out of their world. It's the process of growing within, moving beyond what's happened to you. So it's actually a journey of an individual. It's not a service or a service model as such. So I think the challenge that anyone using mental health services has is of rebuilding a meaningful, valued and satisfying life. And I think the question we have to ask as services is are we doing all that we can to actually enable people to do that? And I think that does rather change the usual focus: typically in services we see ourselves as getting rid of problems or endeavouring to help get rid of someone's problems. I think this is really turning that on its head and saying, how do we help, or did we help, this person to get a life? Maybe we need to be asking different questions about our therapies and treatments. For instance does CBT get you a partner? Does clozapine get you a job? Does occupational therapy get you a home? I think the question is, do we help people in that process of rebuilding their lives and are there different ways that we can be doing that?

**There have been criticisms of recovery, one of the most famous being Recovery in the Bin. What is your opinion on them?**

Which particular criticisms were you thinking of?

**The one that states that recovery is a way to get people through the system and out. There is one quote that I have heard: people are told that ‘Oh, we do recovery model and patients should stay two years and then be discharged back to the GP’.**

The ideas about recovery actually were developed by people who had themselves rebuilt their lives with mental health challenges. People like Judy Chamberlin and Mary O'Hagan. Recovery is not about getting out of services, or time limits on the amount of support you receive. These sorts of ideas come in when ideas about recovery get taken over by commissioners and mental health services and used to their own ends. I would see recovery very much as an ongoing process of growing within and beyond what's happened to you, and that may mean that you need support for a very long time. It may mean that that support comes from services, it may mean that it comes from other places. It may mean more often that you need help from time to time, as we know that many mental health challenges fluctuate. So I think it's really about whether people have access to the support they need when they need it. What I'm talking about is not some throughput model – that is not what recovery is about. I suppose I would say it is very much tied up with some of the social models of disability that have been adopted in the broader disability world, where they talk about people having the right to the support they need in order to do the things that they value in life.

**It seems that there is some kind managerialisation of concepts of recovery and its use to improve throughput. But there's also another dimension. After years in mental health services one thing I have witnessed is the extreme preoccupation with risk. I remember that in one service we were told that we needed to move from a needs-based to a risk-based service. What is your opinion on that and how it fits in with recovery, if ever?**

I certainly find it alarming, that move from the needs-based to a risk-based service. That seems to me bizarre. I don't think that any of us can do anything without taking risks – for example, if you go and talk to someone they may not like you or if you try and get a job you may not get it. Pat Deegan talks a lot about the dignity of risk. Now that's not to say that safety isn't important. But I think what we've got to do is think not about managing risks, but about supporting people to do the things they value as safely as possible. I think we've also got to look at sharing risk. I think the idea that a professional can manage someone's risk is a kind of professional delusion. We are always sharing responsibility for risk, aren't we? The question is what can I do to keep myself safe? What can you do to help me? What can others around do to help me? So I would want to move away from the idea of risk management, to helping people to do things, the things they value as safely as possible. The traditional approaches to risk really do worry me because they tend to be around stopping people doing things, rather than enabling them to do them as safely as they can.

**It's to some degree about balancing the risk and the recovery. Am I right?**

No, I don't think so. I don't think it's a question of balancing risk and recovery. I mean, that rather sounds like something a professional is doing. Every one of us in our lives balances risk against what we want to do. I take a risk every time I drive down to Tooting, but I think it's worth it if I want to go and do my shopping. I think we've got to move away from protecting people, to helping people to do the things that they want to do, the things that matter to them, as safely as possible. And you know, I'm not the only one saying this. I'm sure you've read some of the work of Atul Gawande. In his superb Reith Lectures on the future of medicine, he says we've got it wrong. We think our job is about health and survival. Actually, it's much more than that. It's about well-being. If you look at how he defines well-being, he talks about sustaining people's reasons for wishing to be alive and helping people to pursue their highest life priorities. I think there'd be a great deal to be gained by mental health services looking at that as being their main aim.

**That's interesting. On another topic, one of the things that I really dislike is the way that we are very target driven. For example, we are always pushed to do the clusters. I am not sure how familiar you are with clustering.**

Yes, I have come across clusters! I just find a lot of the ways in which we're thinking about things utterly bizarre. If we think about a person rebuilding their life, whether you want to call it to recovery or not, knowledge of which cluster they fall in seems to be of little value, because individuals differ. They live in different contexts. They live in different communities and different cultures. Their values are different. If we switch the thing around and stop saying our primary aim is to get people better and instead think about helping that person to get a life they value, then I don't see that clusters really help us a great deal in doing this. And I would also say the same about some of our concepts of pathways that sound like a sausage factory to me at times, where you get fed in at one end and fed out at the other. It seems to me we've got to tailor the resources we have to individual people. What are that person's reasons for being alive? What are that person's highest priorities? And I'm not sure clusters of symptomatology or diagnosis, or prescribed pathways, help us in that one iota.

**It is interesting that you mentioned a sausage factory because I call this assembly line psychiatry. The patients are moved along an assembly line of services and things are done to them. And nobody sits with them to take a proper history.**

It is not just the history. It's actually understanding what makes that person tick. You know, those reasons for being alive.

**I do remember that debate you took part in about the way that happiness is overrepresented and it has become industry. And I wanted to hear your opinion now, after years have passed.**

Some have suggested that happiness should be the sort of guiding force of all our social organisational services, etc. I'm not sure that it is the highest human value. I have a lot of climbing books by mountaineers, and I can guarantee you they are not happy in large chunks of climbing Everest or K2 but what they gained from that endeavour was huge. I'm not sure that human development, learning and growth always come from this sort of benign happiness. And I suppose you remember the talk I gave, at the Maudsley. One of the things that really horrified me was when I was involved in closing some of the ghastly old long-stay asylums where people did not even have their own personal clothing. All they had was their bed, their locker and their Gideons Bible immediately beside the next person's bed. The whole thing was regimented – no personal identity whatsoever. And people said they were happy with their lot. I think it's very human. Human beings are amazingly adaptable creatures. And if we put people into the most ghastly situations they adapt to them, but I don't think we can actually call that sort of happiness something which we would want to inspire.

**We can also say that what people want from their lives might not be happiness.**

Quite. And I should say I think it makes you wonder whether all human development and growth comes from some benign kind of happiness. I think we learn as much from the bad bits of our lives as from the good bits. We grow as much from the bad bits as the good bits. And actually, I'm not sure you can have the good bits without the bad bits. How would you know?

**Yeah, absolutely. On a different subject, recently, as you know, we had the Mental Health Act review. And as someone who has been both on the professional side and having lived experience, what do you think of it?**

I was deeply disappointed in it. I believe that it is actively discriminatory against people with mental health challenges on two grounds. I'm going to be quoting here from George Szmukler, because I would be very strongly supportive of his writing on this. First, it means that you have the right to refuse physical healthcare, but you do not have the right to refuse psychiatric treatment. Equally, people with mental health challenges are one group of people you can pre-emptively detain because you may think they may do something – with the possible exception of those defined as terrorists, we don't allow this for any other group of people. We do not lock up a man who drinks because he might go and beat his wife, but we do that with someone who has a mental health problem because they might do something that's a danger to themselves or to other people. So I'm afraid I think we do need some concept of capacity. I think there are issues around capacity legislation, but if you have the capacity to make a judgement about the treatment you receive and you decide against it then you should not be forced to have that treatment. It should be a parallel with physical medicine in that respect.

**And what do you think of advance statements in mental health?**

I have a power of attorney. I think everyone should have a lasting power of attorney. I think that it is possible to say what you would like to happen and what you wouldn't like to happen. But I think the way in which we use them is also important. If you look at research on joint crisis plans, many services couldn't make them work. The staff changed or the plans weren't available at the right time. So in theory, I think it's reasonable to sit down with someone and agree a course of action, should you have problems again. But I think we've got a long way to go in our mental health services before this really is something that can be enacted. If you look at the original work at the Maudsley, it was the only bit of research I've seen that's got a clear indication that you can reduce compulsory detention if joint crisis plans are done properly. They found that they needed someone to assist in that decision-making process, someone neutral to help the team and the individual come to an agreement that both could live with – one that wasn't riding roughshod over the person's wishes. So I think, in theory they are fine, but in practice, I have yet to see them really working effectively in an ordinary clinical setting.

**I just have one last question. I remember that in that debate you talked about ECT and when I came to see you we had a conversation about your experience of it. What do you think about it now?**

I think that each of us has to decide which treatments, interventions, therapies work for us. And for me when I'm profoundly depressed a very short course of ECT is the only thing which actually gets me out of that. However, that doesn't mean that I would market it to other people. I think each of us has to make our own judgements. I don't think I'm in favour of or against drugs, or in favour of or against psychological therapy. I think the real issue is that each person has to work out what is the best way of managing some of the difficulties that they face. And for me, that is one thing I find effective. The important thing is having the power to decide for yourself.
